# Fatigue Is Associated with Anxiety and Lower Health-Related Quality of Life in Patients with Inflammatory Bowel Disease in Remission

**DOI:** 10.3390/medicina59030532

**Published:** 2023-03-09

**Authors:** Tudor Stroie, Carmen Preda, Corina Meianu, Doina Istrătescu, Mircea Manuc, Adina Croitoru, Liana Gheorghe, Cristian Gheorghe, Mircea Diculescu

**Affiliations:** 1 Faculty of Medicine, Carol Davila University of Medicine and Pharmacy, 050474 Bucharest, Romania; 2 Gastroenterology Department, Fundeni Clinical Institute, 022328 Bucharest, Romania; 3 Faculty of Medicine, Titu Maiorescu University, 040441 Bucharest, Romania; 4 Oncology Department, Fundeni Clinical Institute, 022328 Bucharest, Romania

**Keywords:** inflammatory bowel disease, fatigue, quality of life, anxiety, depression

## Abstract

*Background and Objectives:* Inflammatory bowel diseases (IBD) are chronic conditions with an unpredictable course and a remitting–relapsing evolution. Fatigue is a frequent complaint in patients with IBD, affecting approximately half of the newly diagnosed patients with IBD. The aim of this study was to analyze fatigue in patients with IBD in remission. *Materials and Methods:* One hundred nineteen consecutive outpatients diagnosed with IBD for over 3 months that were in corticosteroid-free clinical and biochemical remission at the time of assessment were included in this cross-sectional study. Out of them, 72 (60.5%) were male; the median age was 39 years (IQR 30–47). Seventy-seven patients (64.7%) were diagnosed with Crohn’s disease and forty-two (35.3%) with ulcerative colitis, with a median disease duration of 6 years (IQR 2–10). Fatigue, health-related quality of life (HR-QoL), anxiety and depression were evaluated using the following self-administered questionnaires: FACIT Fatigue, IBDQ 32 and HADS. *Results:* The mean FACIT-Fatigue score was 41.6 (SD ± 8.62), and 38.7% of patients were revealed as experiencing fatigue when a cut-off value of 40 points was used. The mean IBDQ 32 score was 189.4 (SD ± 24.1). Symptoms of anxiety and depression were detected in 37% and 21% of the patients, respectively. In the multivariate analysis, fatigue was significantly associated with lower HR-QoL (OR 2.21, 95% CI: 1.42–3.44, *p* < 0.001), symptoms of anxiety (OR 5.04, 95% CI: 1.20–21.22, *p* = 0.008), female sex (OR 3.32, 95% CI: 1.02–10.76, *p* = 0.04) and longer disease duration (OR 1.13, 95% CI: 1.01–1.27, *p* = 0.04). *Conclusions:* Fatigue is highly prevalent even in patients with inactive IBD and is correlated with lower HR-QoL and anxiety, as well as with clinical factors such as longer disease duration and female sex.

## 1. Introduction

Inflammatory bowel diseases (IBD) with the two subtypes of Crohn’s disease (CD) and ulcerative colitis (UC) represent chronic inflammatory diseases with an unpredictable course, which leads to significant impairment in affected patients’ health-related quality of life (HR-QoL) and high levels of fatigue [1,2].

Fatigue is defined as ‘a persistent, overwhelming sense of tiredness, weakness or exhaustion resulting in a decreased capacity for physical and/or mental work’ [3,4]. It is frequently encountered in chronic inflammatory diseases such as IBD, rheumatoid arthritis, systemic lupus erythematosus or multiple sclerosis; in patients with IBD, fatigue is a common complaint; approximately half of the newly diagnosed patients with IBD experiencing fatigue: 42–47% of patients with UC and 48–62% of patients with CD [5,6]. Regarding the disease activity, the prevalence of fatigue in patients in remission ranges between 41% and 48%; however, in patients with active disease it can be up to 86% [3]. In addition, it is among the most frequent non-intestinal symptoms reported by patients, being more burdensome than gastrointestinal symptoms such as diarrhea or bowel urgency for the IBD patients [7,8]. There is also a strong association between fatigue and HR-QoL, with patients with fatigue having a significantly lower HR-QoL and vice versa [9,10].

Patients with IBD deal with fatigue more often than healthy controls. Active disease, lower hemoglobin values and altered sleeping patterns are among the predictors of fatigue [11].

The etiology of fatigue is multifactorial: chronic inflammatory state, anemia, micronutrients deficiencies due to malabsorption or accelerated intestinal transit, self-imposed dietary restrictions, adverse effects of medication, psychological disturbances, intestinal dysbiosis or dysregulations of the brain–gut axis may play important roles [6].

The management of fatigue in patients with IBD can be quite challenging because of its complex etiology and the limited knowledge on the pathogenesis of IBD-related fatigue, and requires a multidisciplinary approach. Kreijne et al. proposed a fatigue attention cycle that consists of seven steps: screening, assessment of concurrent symptoms, thorough fatigue evaluation, general anti-fatigue strategies, interventions and re-evaluation [12]. Patients with IBD, especially those with active disease, are prone to nutritional and micronutrient deficiencies which should be corrected. A review of the patient’s medication should be performed, since fatigue can be a side effect of certain medications. Non-pharmacological interventions, such as physical activity or psychosocial interventions may be beneficial. Currently, there are no drugs available that could specifically target fatigue in these patients [6,12].

Anxiety and depression are frequently encountered comorbidities in patients with IBD. Up to 35.1% and 21.6% of patients with IBD have symptoms of anxiety and depression, respectively [13]. They play an important role in determining fatigue, being among its most important psychological predictive factors [10]. A study conducted by Norton et al. assessed fatigue in patients with IBD using three different scales for measuring fatigue: Multidimensional Fatigue Inventory (MFI), Inflammatory Bowel Disease Fatigue (IBD-F) and Multidimensional Assessment of Fatigue (MAF). Even though in the univariate analysis both anxiety and depression were predictive factors for fatigue, in the multivariate analysis only depression along with lower QoL were consistently associated with fatigue on all scales [14]. Similarly, a population-based Norwegian study showed that anxiety, depression and poor sleep quality are associated with fatigue at 20 years after the diagnosis of IBD [15]. Conversely, both symptoms of anxiety and depression can manifest as fatigue, and it may be difficult to distinguish them from IBD-associated fatigue [16].

The majority of studies available in the literature that assess fatigue in patients with IBD are not specially focused on patients in remission. Moreover, studies that are designed for patients in remission assess disease activity only on a clinical basis, without considering biochemical markers of disease activity such as CRP and fecal calprotectin.

The aim of this study was to analyze fatigue in patients with IBD in both clinical and biochemical remission and to identify factors associated with it.

## 2. Materials and Methods

### 2.1. Study Population and Design

This was a cross-sectional, observational study.

All patients included in this study were at least 18 years old and were in corticosteroid-free clinical and biochemical remission for more than three months. Pregnant females, patients being treated with corticosteroids, patients with ostomy, perianal disease, extraintestinal manifestations or other significant comorbidities or medical conditions that could have influenced their level of fatigue or quality of life were excluded. Patients already diagnosed with anxiety, depression or other psychiatric conditions were also excluded.

One hundred and forty-one consecutive outpatients diagnosed with IBD for over 3 months that fulfilled the eligibility criteria at the time of assessment were invited to participate in the study. Out of them, 130 accepted and provided their consent to be included in this study. Eight patients had incomplete data and were excluded from the analysis. Another three patients had proof of endoscopic activity and were not considered eligible. Therefore, 119 patients were effectively enrolled in the study.

The patients were treated for IBD at Fundeni Clinical Institute, a tertiary gastroenterology center in Bucharest, Romania. They were invited to participate in this study when they had their routine follow-up visit or treatment administration visit in the outpatient clinic. The enrollment took place between September 2022 and November 2022.

### 2.2. Questionnaires and Data Collection

The participants answered a series of auto-administered questionnaires evaluating their level of fatigue, their HR-QoL and their experiences of anxiety and depression: FACIT Fatigue, IBDQ 32 and HADS. Validated Romanian versions of these questionnaires were used.

The Functional Assessment of Chronic Illness Therapy—Fatigue (FACIT-Fatigue) is a unidimensional scale used to assess fatigue, validated in the general population and a series of chronical conditions, including IBD. The total score ranges from 0 to 52 points, with lower scores representing greater fatigue [17].

The Inflammatory Bowel Disease Questionnaire (IBDQ) is a 32-item questionnaire developed by Guyatt et al. in 1989 used to assess the HR-QoL in patients with IBD. The total score ranges from 0 to 224 points, with higher scores representing better HR-QoL [18].

Hospital Anxiety and Depression Scale (HADS) is a questionnaire used to assess patients for clinically significant anxiety and depression. A total score over 7 points is considered pathological for both anxiety and depression [19,20,21,22].

Fatigue was considered for FACIT-Fatigue score ≤ 40. Symptoms of anxiety and/or depression were considered to be present when patients had a HADS-A score > 7 and/or a HADS-D score > 7, respectively.

Results from blood samples collected during the visit at the hospital were used in this study. Male patients that had a hemoglobin level < 13 g/dL and female patients with a hemoglobin level < 12 g/dL were considered to have anemia.

Data regarding the patients’ demographics, lifestyle and the characteristics of the disease were collected during a short interview and from the patients’ records.

### 2.3. Assessment of Disease Activity

Disease activity was assessed using the Harvey Bradshaw Index (HBI) for CD and Simple Clinical Colitis Activity Index (SCCAI) for UC. Remission was defined for a HBI score ≤ 4 points and SCCAI score ≤ 1 point. In addition, all patients had a CRP level lower than 5 mg/L at inclusion and last fecal calprotectin (within the last 6 months) below 150 ug/g.

### 2.4. Statistical Analysis

Statistical analysis of the data was performed using R version 4.1.2 (1 November 2021) (^©^2021 The R Foundation for Statistical Computing).

In the univariate analysis, *t* test, Chi-squared test and Fisher’s exact test were used to determine whether there were significant differences between patients presenting with fatigue and those without fatigue. Variables were selected taking into account the pre-existing data in the literature [6,10,11,14,15,23,24,25,26,27,28,29,30,31].

Afterwards, a logistic regression model was determined using fatigue as the binary dependent variable and the variables that had a *p* value < 0.2 in the univariate analysis as predictors.

The statistical significance was considered as *p* < 0.05.

## 3. Results

Out of the 119 patients that were included in this study, 72 (60.5%) were male; the median age was 39 years (IQR 30–47). Seventy-seven patients (64.7%) were diagnosed with CD and forty-two (35.3%) with UC, with a median disease duration of 6 years (IQR 2–10). The median duration of remission was 23.5 months (IQR 10–32.5).

The characteristics of the study population, such as demographic data, data regarding the treatment and the disease as well as the prevalence of symptoms of anxiety and depression and the assessment of the HR-QoL are depicted in Table 1.

The vast majority of the patients (88.2%) were following a treatment with a biological agent at the time of inclusion, most of them with infliximab (50.5%) and vedolizumab (32.4%); a smaller proportion of patients were treated with adalimumab (12.3%) and ustekinumab (4.8%); and 11.8% of the patients were following a conventional treatment, either with 5-ASA or azathioprine. Regarding the exposure to previous biological agents, 23.5% of the patients had more than one biological treatment (they had at least one switch). Thirty-seven patients (31.1%) had a history of IBD-related surgery (they underwent at least one IBD-related surgical intervention). One-third of the patients (34.5%) were active smokers.

Regarding the level of education, 6.7% of patients had a low level of education (they completed only the primary cycle of education), 40.3% had a medium level (they completed high-school studies) and 52.9 had a high level of education (they graduated from university or they had a master’s degree or PhD).

Anemia was identified in 14.3% of patients, all of them having only a mild form.

The mean FACIT-Fatigue score was 41.6 (SD ± 8.62), and 38.7% of patients were revealed as having experienced fatigue when a cut-off value of 40 points was used (they had a FACIT-Fatigue score ≤ 40). The mean IBDQ 32 score was 189.4 (SD ± 24.1). Symptoms of anxiety and depression were detected in 37% and 21% of the patients, respectively.

Endoscopic evaluation was not mandatory for inclusion in the study. However, three patients were excluded due to proven endoscopic activity in spite of clinical and biochemical remission. Overall, 40 patients (33.6%) had at least one endoscopic evaluation within the last year. Out of them, 21 had UC and 19 CD. Regarding the patients with UC, 13 underwent rectosigmoidoscopies, and 8 had complete colonoscopies. All patients with CD had ileocolonoscopies. All patients were in endoscopic remission at their last evaluation.

Twenty-eight patients with CD (36.4% of CD patients) underwent CT/MRI enterography or bowel ultrasound within the last year. Out of them, two had small bowel strictures, without dilatation of small bowel loops. The strictures were clinically asymptomatic. Both patients had normal ileocolonoscopies.

### 3.1. Univariate Analysis

Fatigue as assessed by the FACIT-Fatigue score was evaluated in different categories of patients (Table 2).

Female sex was associated with higher levels of fatigue, with 57.4% of female patients experiencing fatigue (*p* = 0.001). Regarding the disease duration, patients affected by fatigue had a significantly longer disease duration (8.9 vs. 6.1 years, *p* = 0.04). Patients with a low level of education also seemed to experience fatigue more frequently; however, the number of patients with a low level of education included in this study was low.

Exposure to a higher number of biological agents was associated with higher levels of fatigue: 64.3% of patients exposed to more than one biological therapy were affected by fatigue (*p* = 0.003). Another factor associated with fatigue was anemia: the majority of patients diagnosed with anemia also experienced fatigue (70.6%, *p* = 0.005). Anxiety and depression strongly influenced the level of fatigue, with a significantly higher proportion of patients with symptoms of anxiety (79.5%, *p* < 0.001) or depression (88%, *p* < 0.001) having fatigue.

Health-related QoL, as measured by IBDQ 32 score, was strongly correlated with the level of fatigue, with patients with fatigue having significantly lower scores (168.5 vs. 202.6 points, *p* < 0.001).

Age, disease phenotype, smoking status, employment status and history of IBD-related surgery were not significantly associated with fatigue in our sample of patients.

### 3.2. Multivariate Analysis

A logistic regression model was determined by analyzing all variables that had a *p* value < 0.2 in the univariate analysis.

Fatigue was significantly associated with lower HR-QoL (OR 2.21, 95% CI: 1.42–3.44, *p* < 0.001), symptoms of anxiety (OR 5.04, 95% CI: 1.20–21.22, *p* = 0.008), female sex (OR 3.32, 95% CI: 1.02–10.76, *p* = 0.04) and with longer disease duration (OR 1.13, 95% CI: 1.01–1.27, *p* = 0.04) (Table 3).

Other variables such as depression, disease phenotype, lower level of education, the use of multiple biological therapies and anemia were not significantly associated with fatigue in the multivariate analysis.

## 4. Discussion

This is, to our knowledge, the first study designed to evaluate fatigue in patients with IBD in remission in Romania. It identified several factors associated with fatigue in a population of patients that are considered to be optimally treated, without the need of treatment optimization or surgical procedures, and it highlights the fact that even these patients still have to deal with high levels of fatigue. The early detection of fatigue and acting on factors that are associated with it could lead to a significant improvement in patients’ HR-QoL and psychosocial status.

As our study shows, fatigue is a common symptom in patients with IBD in remission, identified in up to 38.7% of the patients in our cohort. While other studies report similar results [27,28], this percentage is, however, lower compared with data presented in a study conducted by Villoria et al. that also assessed fatigue using the FACIT-Fatigue score with the same cut-off value of 40 points. In this study conducted on 202 outpatients with inactive IBD, fatigue was present in 54% of the patients; this may be explained by the fact that the eligibility criteria in our study were more restrictive, and patients were also in biochemical remission [10].

In our study population, fatigue was significantly associated with lower HR-QoL, which is also confirmed by other studies [14,27,29,30,32]. However, the majority of them are not focused on patients in remission. We identified a significant association between fatigue and lower HR-QoL: patients with fatigue had significantly lower IBDQ 32 scores: 168.5 vs. 202.6 points (*p* < 0.001), which was also significant in the multivariate analysis.

Depression seems to be highly correlated with fatigue, as reported by multiple studies in the literature [14,23,29,30,31]. Conversely, only a few of them report anxiety to be significantly associated with fatigue in patients with inflammatory bowel disease, and even fewer for patients in remission. In our study, even if they were both significantly associated with fatigue in the univariate analysis, in the multivariate analysis only anxiety was significantly associated with fatigue (OR 5.04, 95% CI: 1.20–21.22, *p* = 0.008).

Female sex is a well-known factor associated with fatigue [14,24,25,28,29,33]. Similar to general population, in our group of patients, fatigue was strongly associated with female sex. This association is confirmed by several other studies performed on IBD patients [7,27,28,31,34].

Patients with a longer disease duration may have experienced a more protracted course and may have had multiple relapses or surgical interventions. In our study, this group of patients was affected more frequently by fatigue. Conversely, other studies report lower levels of fatigue in patients with longer disease durations, which could be explained by the adjustment to the chronic disease over time [28].

Fatigue was not associated with previous IBD-related surgeries. Even if there are studies in the literature reporting that IBD-related surgery is associated with fatigue [26], other studies did not find a significant association [10,30], and others report an improvement of fatigue and HR-QoL after surgery [35,36]. Patients that had bowel strictures and underwent surgical resection may have an improved food-related quality of life and may be less prone to restrictive dietary behavior due to the alleviation of obstructive symptoms [37]. In addition, patients with ileal resection were systematically assessed for vitamin B12 deficiency and supplemented if necessary, which may have contributed to the improvement of fatigue in our study.

Anemia, even if it was significantly associated with fatigue in the univariate analysis, was not significant in the multivariate analysis. However, data reported in the literature are divergent. While some studies report the lack of association between fatigue and anemia or iron deficiency in patients with inactive disease [7,38], others found an association between hemoglobin levels and fatigue [6,23,27].

Vitamin and micronutrients deficiencies may play an important role in the etiology of fatigue [6]. Although they were not specifically assessed in this study, as previously mentioned, vitamin B12 level was systematically assessed in patients with ileal resection as part of the IBD management. Fifteen patients had ileal resection; out of them, six developed vitamin B12 deficiency and received supplementation. Similarly, patients with anemia were assessed for iron, vitamin B12 and folic acid deficiencies. All patients identified with deficiencies received supplementations accordingly.

Even though the vitamin D level was not routinely assessed, an important proportion of patients treated in our center use vitamin D supplements. However, the association between fatigue and vitamin D deficiency in patients with IBD is controversial. While some studies found an association between muscle fatigue and lower vitamin D level [39], others report that vitamin D deficiency is not associated with fatigue in patients with IBD [30,40,41].

### Strengths and Limitations

The study analyzed fatigue in patients with IBD in both clinical and biochemical remission, in correlation with anxiety, depression and HR-QoL. It was conducted on a population of patients that were considered to be optimally treated. Several factors associated with fatigue were identified, such as lower HR-QoL, anxiety, female sex and longer disease duration.

On the other hand, it has several limitations, such as the inability to establish the causality due to its cross-sectional design; disease remission was evaluated on clinical and biochemical bases, without colonoscopies performed at inclusion. Given the fact that this was a single-center study and the prevalence of IBD in Romania is low [42], the sample size was limited. Another possible limitation is that the patients were treated in a tertiary gastroenterology center and because of this fact they may have had a more severe disease.

## 5. Conclusions

In summary, this study showed that even in patients with inactive IBD there is a high prevalence of fatigue. In addition, it identified factors that are significantly associated with fatigue: lower HR-QoL, anxiety, as well as other clinical factors such as longer disease duration and female sex.

Because fatigue is a multifactorial and complex condition, further research with larger groups of patients and multicenter trials should focus on identifying other predictive or etiological factors for fatigue in patients with IBD.

## Figures and Tables

**Table 1:** Characteristics of the study population.

Characteristic		
Sex	Male, n (%) Female, n (%)	72 (60.5%) 47 (39.5%)
Median age, years (IQR)		39 (30–47)
Phenotype	Crohn’s disease, n (%) Ulcerative colitis, n (%)	77 (64.7%) 42 (35.3%)
Median disease duration, years (IQR)		6 (2–10)
Active smoking, n (%)		41 (34.5%)
Treatment	Biologic, n (%) Conventional, n (%)	105 (88.2%) 14 (11.8%)
Type of biological treatmen	Infliximab, n (%) Adalimumab, n (%) Vedolizumab, n (%) Ustekinumab, n (%)	53 (50.5%) 13 (12.3%) 34 (32.4%) 5 (4.8%)
Number of biological treatments	0–1, n (%) >1, n (%)	91 (76.4%) 28 (23.5%)
History of IBD-related surgery, n (%)		37 (31.1%)
Anemia, n (%)		17 (14.3%)
Level of education	Low, n (%) Medium, n (%) High, n (%)	8 (6.7%) 48 (40.3%) 63 (52.9%)
Currently employed, n (%)		100 (84%)
FACIT-Fatigue	Mean (± SD) FACIT-F ≤ 40, n (%)	41.6 (±8.62) 46 (38.7%)
Mean IBDQ score (± SD)		189.4 (±24.1)
Symptoms of anxiety, n (%)		44 (37%)
Symptoms of depression, n (%)		25 (21%)

**Table 2:** Univariate analysis of factors associated with fatigue.

	Fatigue (FACIT Fatigue ≤ 40)	Non-Fatigue (FACIT Fatigue > 40)	Significance (*p*)
Mean age, years (±SD)	39.1 (13.1)	40.2 (12.2)	0.66
Female sex, n (%)	27 (57.4)	20 (42.6)	0.001
CD Phenotype, n (%)	34 (44.2)	43 (55.8)	0.14
Mean disease duration, years (±SD)	8.9 (8.4)	6.1 (4.9)	0.04
Active smoking, n (%)	16 (39)	25 (61)	0.86
Lower level of education, n (%)	6 (75)	2 (25)	0.04
>1 biological therapy, n (%)	18 (64.3)	10 (35.7)	0.003
Unemployment, n (%)	8 (42.1)	11 (57.9)	0.86
Anemia, n (%)	12 (70.6)	5 (29.4)	0.005
History of IBD-related surgery, n (%)	16 (43.2)	21 (56.8)	0.62
Symptoms of anxiety, n (%)	35 (79.5)	9 (20.5)	<0.001
Symptoms of depression, n (%)	22 (88)	3 (12)	<0.001
HR-QoL, mean IBDQ32 (±SD)	168.5 (21.4)	202.6 (14.3)	<0.001

**Table 3:** Multivariate analysis of factors associated with fatigue in univariate analysis.

	OR of Fatigue (95% CI)	Significance (*p*)
Female sex	3.32 (1.02–10.76)	0.04
CD Phenotype	4.67 (0.89–24.37)	0.11
Longer disease duration *	1.13 (1.01–1.27)	0.04
Lower level of education	4.05 (0.27–60.01)	0.30
>1 biological therapy	1.13 (0.24–5.31)	0.87
Anemia	5.16 (0.54–48.45)	0.15
Symptoms of anxiety	5.04 (1.20–21.22)	0.008
Symptoms of depression	1.98 (0.32–11.99)	0.45
Lower HR-QoL ^†^	2.21 (1.42–3.44)	<0.001

* one year increase, ^†^ ten points decrease in IBDQ score.

## Data Availability

The data presented in this study are available on request from the corresponding author.
